# Factor structure of the Singapore English version of the KINDL^® ^children quality of life questionnaire

**DOI:** 10.1186/1477-7525-5-4

**Published:** 2007-01-19

**Authors:** Hwee-Lin Wee, Ulrike Ravens-Sieberer, Michael Erhart, Shu-Chuen Li

**Affiliations:** 1 Department of Pharmacy, National University of Singapore, Republic of Singapore; 2 Robert Koch Institute, Berlin, Germany; 3 Discipline of Pharmacy and Experimental Pharmacology, School of Biomedical Sciences, Faculty of Health, University of Newcastle, Callaghan, Australia

## Abstract

**Background:**

Quality of life (QoL) outcomes are useful in the assessment of physical, mental and social well-being and for informed healthcare decision making. However, few studies have evaluated QoL issues among Asian children due largely to the lack of culturally valid and reliable QoL questionnaires. Hence, we aimed to report the psychometric properties, in particular factor structure, of KINDL (Singapore) questionnaires among school-going children.

**Methods:**

Students aged 8–16 years from participating schools were selected by convenience sampling. Subjects self-completed KINDL-Kid (Singapore) and KINDL-Kiddo (Singapore) questionnaires, which were cross-culturally adapted from KINDL (Germany English) for use in Singapore. We evaluated floor and ceiling effects, internal consistency and performed factor analysis.

**Results:**

A total of 328 respondents (mean (SD) age: 9.6 (1.31) years; 67% female; 75% Chinese, 16% Malays, 9% Indians and others) completed KINDL-Kid while 1,026 respondents (mean (SD) age: 14.0 (1.00) years; 82% female; 82% Chinese, 12% Malays; 6% Indians and others) completed KINDL-Kiddo. Mean (SD) TOTAL QoL score was 65.5 (12.76) and 56.6 (11.92) for KINDL-Kid and KINDL-Kiddo, respectively. Floor and ceiling effects were important in five of six KINDL-Kid and two of six KINDL-Kiddo subscales. Reliability coefficients ranged from 0.40 to 0.71 (KINDL-Kid) and 0.44 to 0.84 (KINDL-Kiddo). Factor analysis generated eight and seven factors in KINDL-Kid and KINDL-Kiddo, respectively.

**Conclusion:**

KINDL-Kiddo exhibited good psychometric properties and may be used to assess QoL in this multi-ethnic English-speaking Asian population. However, psychometric properties of KINDL-Kid may need to be improved either by developing new items or modifying existing items.

## Background

Worldwide, there is an increasing interest in the measurement of quality of life (QoL) outcomes, which are useful in the assessment of physical, mental and social well-being and are potentially useful for informing healthcare decision making [1,2]. However, QoL assessments within Asian contexts have been limited due to the lack of questionnaires with demonstrated validity and reliability among Asians and the problem is particularly acute in the Asian pediatric population.

Although several children QoL questionnaires have been developed [3], very few were culturally adapted and validated for use in Asia. At the same of this study (2003), we identified only three questionnaires (in Arabic, Korean, Hebrew and Turkish) that were validated in Asian pediatric populations – Child Health Questionnaire, Childhood Health Assessment Questionnaire, and Quality of Life in Childhood Cancer [4-8]. However, only one of these questionnaires is a generic (Child Health Questionnaire) QoL questionnaire while the others are disease-specific questionnaires which have limited applications for the general pediatric population.

Furthermore, these language versions were not used in most countries in Asia, in particular Southeast Asia. Hence, we undertook a study to evaluate the psychometric properties of KINDL^® ^– a generic children's quality of life questionnaire among English-speaking Singaporeans [9]. English was chosen because it is a universal language with potentially wide application in several Asian countries, including Hong Kong, the Philippines and Malaysia.

It was encouraging that since our first study was published [1], several more QoL questionnaires became available for use in the Asian pediatric population [10-13]. A few other QoL validation studies among Asians [14-17] were also published at around the same time as our study. However, only two of these studies evaluated factor validity of the questionnaire used [13,18]. In addition, language availability remains a problem. In the case of Singapore, as many as 11% (44,756 of 412,796) of Singaporeans aged 15–24 understood English only [19]. Hence, an English version of a generic QoL questionnaire is needed if such studies are to be representative of the general pediatric population.

In cross-cultural adaptation and translation of QoL questionnaires, it is important to ensure that psychometric properties of the original questionnaire are preserved in the new cultural setting. In our earlier study among children aged 8–16 years [9], internal consistencies of KINDL-Kid was 0.75 and that of KINDL-Kiddo was 0.84. The general accepted Cronbach's alpha for group level comparison was >= 0.5 [20,21] and that for person level comparison was >= 0.8 [21]. Hence, our results showed that the KINDL questionnaires were satisfactory for group level comparison and less satisfactory for person level comparison. This might suggest the translated KINDL questionnaire might be psychometrically different from the source English version. We aimed to confirm this hypothesis using a large school-based sample (who are likely to be representative of the general pediatric population). Thus, the aim of this paper is to evaluate the psychometric properties, in particular, factor structure of the Singapore English version of KINDL questionnaires among school-going children.

## Methods

### Study design and subject recruitment

This study was conducted over several days in March 2002. Permission to conduct the study in school children was obtained from the Ministry of Education, Singapore. Invitations for participation were sent via either facsimile or e-mail to primary and secondary schools (equivalent to the junior and high schools in the United States). A follow-up facsimile or e-mail was sent a week after the first mailer stating the study aims and design. Further details were provided to those principals who agreed to participate in the study.

Students aged 8–16 years old were selected by convenience sampling by the teacher in-charge of the survey. The aim was to minimize administrative burden so as to encourage participation. As English is the primary medium of teaching in Singapore, subjects completed the Singapore English versions of KINDL questionnaires and a simple demographic information sheet by self-completion without any assistance.

### Instrument

The Singapore English versions of KINDL questionnaires were cross-culturally adapted [9] from the source Germany English versions [22] (available with permission from the developers), using standard guidelines, including independent forward and back translation and cognitive debriefing in focus groups. KINDL questionnaire is available in three age versions: KINDL-Kiddy (4–7 years), KINDL-Kid (8–12 years) and KINDL-Kiddo (13–16 years) with both parents and self-reports. In this study, we used the self-report versions of KINDL-Kid and KINDL-Kiddo. Each KINDL questionnaire comprised 24 items (with five answer categories) yielding a general QoL score (TOTAL) and six subscales: physical health (PH), general health (GH), family functioning (FAM), self-esteem (PER), social functioning (FREN) and school functioning (SCH) scores (Table 1). Reverse scoring was applied to some items so that higher item scores represent better QoL. Item scores were summed up to give subscale scores, and subscale scores summed up to give a TOTAL score. The raw score was transformed to a scale of 0–100 to facilitate interpretation.

**Table 1:** Items in KINDL-Kid and KINDL-Kiddo

**KINDL-Kid**	**KINDL-Kiddo**
**Physical Health Scale**	**Physical Health Scale**
PH1. ... felt ill	PH1. ... felt ill
PH2. ... headache or tummy-ache	PH2. ... in pain
PH3. ... tired and sleepy	PH3. ... tired and sleepy
PH4. ... strong and full of energy	PH4. ... felt strong and full of energy
	
**General Health Scale**	**General Health Scale**
GEN1. ... had fun and laughed a lot	GEN1. ... had fun and laughed a lot
GEN2. ... bored	GEN2. ... was bored
GEN3. ... felt alone	GEN3. ... felt alone
GEN4. ... scared	GEN4. ... was scared or unsure of myself
	
**Self-esteem Scale**	**Self-esteem Scale**
PER1. ... proud of myself	PER1. ... proud of myself
PER2. ... felt on top of the world	PER2. ... felt on top of the world
PER3. ... felt pleased with myself	PER3. ... felt pleased with myself
PER4. ... had lots of good ideas	PER4. ... had lots of good ideas
	
**Family Functioning Scale**	**Family Functioning Scale**
FAM1. ... got on well with my parents	FAM1. ... got on well with my parents
FAM2. ... felt fine at home	FAM2. ... felt fine at home
FAM3. ... quarrelled at home	FAM3. ... quarrelled at home
FAM4. ... stopped from doing certain things	FAM4. ... felt restricted by my parents
	
**Social Functioning Scale**	**Social Functioning Scale**
FREN1. ... played with friends	FREN1. ... did things together with my friends
FREN2. ... other kids liked me	FREN2. ... was a "success" with my friends
FREN3. ... got along well with my friends	FREN3. ... got along well with my friends
FREN4. ... felt different from other children	FREN4. ... felt different from other people
	
**School Functioning Scale**	**School Functioning scale**
SCH1. ... doing my school work was easy	SCH1. ... doing my school work was easy
SCH2. ... enjoyed my lessons	SCH2. ... found school interesting
SCH3. ... looked forward to the weeks ahead	SCH3. ... worried about my future
SCH4. ... was afraid of bad marks or grades	SCH4. ... was worried about getting bad marks or grades

### Statistical analyses

The number of responses at minimum (floor) and maximum (ceiling) scores were reported. Internal consistencies of individual subscales were evaluated using Cronbach's alpha. Floor and ceiling effects exceeding 15% have been considered high [23]. In our study, however, we applied a more stringent criteria with floor and ceiling effects exceeding 5% considered as high. Factor structure of KINDL-Kid (Singapore) and KINDL-Kiddo (Singapore) were evaluated using principal component analysis with varimax rotation. The criterion chosen to determine that an extracted factor accounted for a reasonably large proportion of the total variance was based on an eigenvalue greater than 1. There is no consensus on the minimum sample size for factor analysis, with recommendations ranging from 100 to 300 [24,25]. We hypothesised that KINDL (Singapore) will retain the same factor structure (Table 1) as the source KINDL (Germany). All statistical analyses were performed using STATA [26].

## Results

### Subjects

A total of 181 e-mail and faxes was sent with follow-up e-mail or faxes. In response, three primary (one girl's school, two co-ed) and five secondary schools (one boy's school, one girl's, three co-ed) agreed to participate. The most common reason that principals gave to decline the invitation was that their students were already involved in other studies. A total of 328 respondents (mean (standard deviation, SD) age: 9.6 (1.31) years; 67% female; 75% Chinese, 16% Malays, 9% Indians and others) completed KINDL-Kid (Singapore) while 1,026 respondents (mean (SD) age: 14.0 (1.00) years; 82% female; 82% Chinese, 12% Malays, 6% Indians and others) completed KINDL-Kiddo (Singapore). There were no missing data.

### Data analyses

#### Mean, Standard deviation (SD), Floor and Ceiling Effects (Table 2)

**Table 2:** Score distributions, floor and ceiling responses and internal consistency of KINDL-Kid (Singapore) and KINDL-Kiddo (Singapore) subscales

**KINDL-Kid Subscale**	**Mean (SD)**	**Range**	**Floor Responses (N, %)**	**Ceiling Responses (N, %)**	**Internal Consistency**^†^
**TOTAL**	65.5 (12.76)	31.3 to 100	0	2 (0.6)	0.79
PH	75.1 (17.08)	12.5 to 100	0	53 (16.2)	0.49
GEN	76.7 (17.47)	12.5 to 100	0	39 (11.9)	0.50
PER	44.0 (25.10)	0 to 100	24 (7.3)	12 (3.7)	0.71
FAM	71.9 (18.56)	25 to 100	0	36 (11.0)	0.46
FREN	66.2 (20.79)	12.5 to 100	0	22 (6.7)	0.52
SCH	59.0 (20.32)	0 to 100	3 (0.9)	18 (5.4)	0.40
					

**KINDL-Kiddo Subscale**	**Mean (SD)**	**Range**	**Floor Responses (N, %)**	**Ceiling Responses (N, %)**	**Internal Consistency**^†^

**TOTAL**	56.6 (11.92)	3.1 to 95.8	0	0	0.83
PH	61.4 (16.66)	0 to 100	3 (0.3)	9 (0.9)	0.62
GEN	66.8 (17.36)	0 to 100	2 (0.2)	28 (2.7)	0.63
PER	39.7 (22.69)	0 to 100	57 (5.6)	35 (3.4)	0.84
FAM	68.3 (20.93)	0 to 100	3 (0.3)	76 (7.4)	0.76
FREN	62.2 (17.53)	0 to 100	4 (0.4)	21 (2.1)	0.61
SCH	41.4 (16.57)	0 to 100	18 (1.8)	3 (0.3)	0.44

Abbreviations: TOTAL = Total QoL Scores; PH = Physical health; GEN = General health; PER = Self-esteem; FAM = Family functioning; FREN = Social functioning; SCH = School functioning.

^†^Internal consistency was measured using Cronbach's alpha.

Mean (SD) of the TOTAL QoL score was 65.5 (12.76) and 56.6 (11.92) for KINDL-Kid (Singapore) and KINDL-Kiddo (Singapore), respectively. For KINDL-Kid (Singapore), floor effect was negligible for all subscales except PER, where number of response at minimum scores was 7.3%. Ceiling effect was more pronounced with all subscales except PER having ceiling responses exceeding 5%. For KINDL-Kiddo (Singapore), similar to KINDL-Kid (Singapore), floor effect was negligible for all subscales except PER, where floor response was 5.6%. However, ceiling effect was less pronounced than in KINDL-Kid (Singapore), with only one (FAM) of six subscales having ceiling responses that exceeded 5%.

#### Internal consistency (Table 2)

KINDL-Kid (Singapore): Internal consistency as measured by Cronbach's alpha was 0.79 for KINDL TOTAL and ranged from 0.40 (SCH) to 0.71 (FAM) for the subscales. None of the scales had internal consistencies that were considered acceptable for person level comparisons. Overall these internal consistencies were lower than the alphas reported for the original German version (alpha = 0.63 to 0.84), which however were calculated across both KINDL-Kid and KINDL-Kiddo [27].

KINDL-Kiddo (Singapore): The overall internal consistency of KINDL-Kiddo (Singapore) was higher than KINDL-Kid (Singapore). Cronbach's alpha was 0.83 for KINDL TOTAL with Cronbach's alpha ranging from 0.44 (SCH) to 0.84 (PER) for the subscales. Internal consistencies of the TOTAL and the subscales PER and FAM were considered acceptable for person level comparisons. Some internal consistencies were lower than the alphas reported for the original German version (alpha = 0.63 to 0.84) [27], while others were higher.

**Table 3:** Factor structure of KINDL-Kid (Singapore) and KINDL-Kiddo (Singapore) questionnaires

KINDL-Kid subscale	Factor loading*								KINDL-Kiddo subscale	Factor loading*						
	1	2	3	4	5	6	7	8		1	2	3	4	5	6	7

PH1					-0.80				PH1						0.85	
PH2					-0.78				PH2						0.70	
PH3					-0.47				PH3						0.44	
PH4	0.47		0.47						PH4						0.41	
GEN1				0.48					GEN1			0.60				
GEN2						-0.86			GEN2					-0.70		
GEN3						-0.80			GEN3					-0.72		
GEN4		0.54							GEN4					-0.62		
PER1			0.70						PER1	0.85						
PER2			0.71						PER2	0.83						
PER3			0.71						PER3	0.82						
PER4			0.54						PER4	0.69						
FAM1	0.75								FAM1		0.80					
FAM2	0.68								FAM2		0.74					
FAM3								0.72	FAM3		0.70					
FAM4		0.57							FAM4		0.68					
FREN1			0.81						FREN1			0.84				
FREN2			0.63						FREN2			0.75				
FREN3			0.69						FREN3			0.76				
FREN4								0.71	FREN4					-0.47		
SCH1							0.68		SCH1							0.73
SCH2							0.63		SCH2							0.74
SCH3	0.44						0.50		SCH3				0.83			
SCH4		0.72							SCH4				0.86			

* Only factor loadings above 0.40 are shown.

#### Factor analysis (Table 3)

KINDL-Kid (Singapore): KINDL-Kid (Singapore) items did not load onto the six factors originally hypothesised. Instead, eight factors were generated. The cumulative percentage of total variance explained by the eight-factor solution was 60.4%. A six-factor solution would have explained 51.3% of the total variance. One of the new factors comprised of GEN4, FAM4 and SCH4, items that were originally from three different subscales (Factor 2, Table 4). In addition, some items were reorganized and loaded onto subscales different from those originally hypothesised. For example, GEN1 loaded onto FREN instead of GEN (Factor 5, Table 4).

**Table 4:** Distribution of items based on factor structure of KINDL-Kid (Singapore) and KINDL-Kiddo (Singapore) questionnaires

**KINDL-Kid**	**KINDL-Kiddo**
**Factor 1**	**Factor 1**
PH1. ... felt ill	PH1. ... felt ill
PH2. ... headache or tummy-ache	PH2. ... in pain
PH3. ... tired and sleepy	PH3. ... tired and sleepy
	PH4. ... felt strong and full of energy
	
**Factor 2**	**Factor 2**
GEN4. ... scared	GEN2. ... was bored
FAM4. ... stopped from doing certain things	GEN3. ... felt alone
SCH4. ... was afraid of bad marks or grades	GEN4. ... was scared or unsure of myself
	FREN4. ... felt different from other people
	
**Factor 3**	**Factor 3**
PER1. ... proud of myself	PER1. ... proud of myself
PER2. ... felt on top of the world	PER2. ... felt on top of the world
PER3. ... felt pleased with myself	PER3. ... felt pleased with myself
PER4. ... had lots of good ideas	PER4. ... had lots of good ideas
PH4. ... strong and full of energy	
	
**Factor 4**	**Factor 4**
FAM1. ... got on well with my parents	FAM1. ... got on well with my parents
FAM2. ... felt fine at home	FAM2. ... felt fine at home
PH4. ... strong and full of energy	FAM3. ... quarrelled at home
	FAM4. ... felt restricted by my parents
	
**Factor 5**	**Factor 5**
FREN1. ... played with friends	FREN1. ... did things together with my friends
FREN2. ... other kids liked me	FREN2. ... was a "success" with my friends
FREN3. ... got along well with my friends	FREN3. ... got along well with my friends
GEN1. ... had fun and laughed a lot	GEN1. ... had fun and laughed a lot
	
**Factor 6**	**Factor 6**
SCH1. ... doing my school work was easy	SCH1. ... doing my school work was easy
SCH2. ... enjoyed my lessons	SCH2. ... found school interesting
SCH3. ... looked forward to the weeks ahead	
	
**Factor 7**	**Factor 7**
GEN2. ... bored	SCH3. ... worried about my future
GEN3. ... felt alone	SCH4. ... was worried about getting bad marks or grades
	
**Factor 8**	
FAM3. ... quarrelled at home	
FREN4. ... felt different from other children	

KINDL-Kiddo (Singapore): Results of factor analysis was more reassuring for KINDL-Kiddo (Singapore) compared with KINDL-Kid (Singapore). Seven factors were identified (eigenvalue > 1.0). The cumulative percentage of total variance explained by the seven-factor solution was 62.2%. A six-factor solution would have explained 57.8% of the total variance. Factors 1, 3 and 4 (Table 4) of KINDL-Kid (Singapore), corresponded exactly to PH, PER and FAM subscales of KINDL-Kiddo (Germany). Factors 2 and 5 of KINDL-Kiddo (Singapore) were almost identical to GEN and FREN subscales of KINDL-Kiddo (Germany) except for an exchange of two items GEN4 and FREN4 between the two subscales. Interestingly, Factors 6 and 7 actually comprised of items (two per factor) that were from the SCH subscale of KINDL-Kid (Germany).

## Discussion

In this study, we evaluated the psychometric properties of KINDL (Singapore) questionnaires among English-speaking Singaporean children and adolescents recruited from schools. The psychometric properties of KINDL-Kiddo (Singapore) in this Asian population were reassuring albeit with a few areas needing improvement. First, internal consistency was good for the TOTAL instrument score. Two subscales displayed problems with regards to floor and ceiling effects. Internal consistency was sometimes insufficient in the subscales, but apart from this was good for group level comparisons. However, we should aim to achieve internal consistency good enough for person level comparisons. Yet, this is technically difficult as respondents were relatively young, and their responses are likely to be less reliable than adults. Arguably the homogeneity of the sample – comprising of healthy children and adolescents only with presumably fewer variation in their QoL trait parameter values – had led to slightly reduced Cronbach alpha values. Second, factor structure of KINDL-Kiddo (Singapore) was almost identical to KINDL-Kiddo (Germany), except for the partitioning of SCH subscale into two factors in KINDL-Kiddo (Singapore). This could be addressed by modifying one or more items in SCH subscale.

The psychometric properties of KINDL-Kid (Singapore) were less reassuring and needs to be improved in several aspects. Otherwise, cross-cultural comparisons of findings made using KINDL-Kid questionnaires could be misleading because the equivalence of the construct measured is questionable. First, ceiling effects needs to be reduced, possibly by including items of greater difficulty. Second, internal consistency needs to be improved by either eliminating items that do not contribute to a subscale or by developing new items with better psychometric properties and/or modifying existing items. Third, we would suggest to further examine the items in KINDL-Kid (Singapore) either by modifying existing items or adding new items such that the factor structure reflects that of the KINDL-Kid (Germany). This is because in a focus group study among Singaporean children and adolescents [27], we found that Singaporean and Western children/adolescents share a remarkably similar notion of general and health-related QoL. Hence, it is theoretically possible for both KINDL-Kid (Singapore) and KINDL-Kid (Germany) to achieve the same factor structure.

We recognized there are limitations in this study. First, our sample is unlikely to be representative of the general pediatric population given the very low participation rate (4.5%) from the school community. Second, at the school level, we opted for convenience sampling in order to improve participation rate. As a result, there was an over-representation of female subjects (general population: 50.5% female [28]) and slight under-representation of the ethnic minorities (general population: 13.9% Malays and 9.3% Indians and others [28]). Nevertheless, the results were encouraging and provided empirical support for further studies to evaluate KINDL (Singapore) questionnaires. Third, Singapore is a multi-ethnic, multi-lingual society but we have used only the English language questionnaires. However, as English is the main language (besides mother tongue) used in all educational institutions, this is unlikely to pose a problem. It should be noted that there were no subjects who had to be excluded from the study because they could not understand English.

## Conclusion

KINDL-Kiddo (Singapore) exhibited good psychometric properties and may be used to assess quality of life in this multi-ethnic English-speaking Asian population. However, the psychometric properties of KINDL-Kid (Singapore) need to be improved. Furthermore, to cater to the multi-lingual sociocultural environment in Singapore and other Asian countries, various language versions will be needed. For the moment, it is strongly recommended to focus on KINDL TOTAL score when interpreting KINDL data.

## List of abbreviations

FAM Family functioning

FREN Social functioning

GEN General health

PER Self-esteem

PH Physical

QoL quality of life

SCH School functioning

SD Standard deviation

TOTAL General QoL score

## Competing interests

The authors declare that they have no competing interests.

## Authors' contributions

HLW conceived the study, participated in the design and coordination of the study and performed the statistical analysis. SCL and URS conceived the study, participated and provided oversight for its design and coordination. ME performed the statistical analysis.
